# Reclassification and subtyping of so-called malignant fibrous histiocytoma of bone: comparison with cytogenetic features

**DOI:** 10.1186/2045-3329-1-10

**Published:** 2011-10-13

**Authors:** Fredrik Mertens, Salvatore Romeo, Judith VMG Bovée, Roberto Tirabosco, Nick Athanasou, Marco Alberghini, Pancras CW Hogendoorn, Angelo P Dei Tos, Raf Sciot, Henryk A Domanski, Kristina Åström, Nils Mandahl, Maria Debiec-Rychter

**Affiliations:** 1 Department of Clinical Genetics, University and Regional Laboratories, Skåne University Hospital, Lund University, Lund, Sweden; 2 Department of Pathology, Treviso Regional Hospital, Treviso, Italy; 3 Department of Pathology, Leiden University Medical Center, Leiden, The Netherlands; 4 Department of Pathology, Royal National Orthopaedic Hospital, London, UK; 5 Department of Pathology, Nuffield Orthopaedic Center, Oxford, UK; 6 Department of Pathology, Rizzoli Orthopaedic Institute, Bologna, Italy; 7 Department of Pathology, Catholic University of Leuven, Leuven, Belgium; 8 Department of Pathology, Lund University Hospital, Lund, Sweden; 9 Department of Pathology, Karolinska Hospital, Stockholm, Sweden; 10 Center for Human Genetics, Catholic University of Leuven, Leuven, Belgium

**Keywords:** Malignant fibrous histiocytoma of bone, chromosome banding, EWSR1, FUS

## Abstract

**Background:**

The diagnostic entity malignant fibrous histiocytoma (MFH) of bone is, like its soft tissue counterpart, likely to be a misnomer, encompassing a variety of poorly differentiated sarcomas. When reviewing a series of 57 so-called MFH of bone within the framework of the EuroBoNeT consortium according to up-to-date criteria and ancillary immunohistochemistry, a fourth of all tumors were reclassified and subtyped.

**Methods:**

In the present study, the cytogenetic data on 11 of these tumors (three myoepithelioma-like sarcomas, two leiomyosarcomas, one undifferentiated pleomorphic sarcoma with incomplete myogenic differentiation, two undifferentiated pleomorphic sarcomas, one osteosarcoma, one spindle cell sarcoma, and one unclassifiable biphasic sarcoma) are presented.

**Results:**

All tumors were high-grade lesions and showed very complex karyotypes. Neither the overall pattern (ploidy level, degree of complexity) nor specific cytogenetic features distinguished any of the subtypes. The subgroup of myoepithelioma-like sarcomas was further investigated with regard to the status of the *EWSR1 *and *FUS *loci; however, no rearrangement was found. Nor was any particular aberration that could differentiate any of the subtypes from osteosarcomas detected.

**Conclusions:**

chromosome banding analysis is unlikely to reveal potential genotype-phenotype correlations between morphologic subtypes among so-called MFH of bone.

## Background

Only some decades ago, malignant fibrous histiocytoma (MFH) was considered the most common soft tissue sarcoma among adults. However, with the introduction of more stringent morphologic and immunohistochemical criteria, it turned out that it was possible to reclassify the vast majority of those tumors as, e.g., poorly differentiated leiomyosarcomas or dedifferentiated liposarcomas [1]. For the few cases in which no signs of differentiation could be discerned, the term undifferentiated pleomorphic sarcoma (UPS) was introduced [2,3]. Importantly, the subclassification of MFH tumors into different lineages of differentiation was shown to be of prognostic significance, with immunohistochemical expression of smooth muscle actin (incomplete myogenic differentiation) being associated with worse outcome [4].

In the latest edition of the WHO classification of soft tissue and bone tumors [2], the new view on MFH tumors was introduced for the soft tissue lesions [3], but not for bone tumors [5]. According to the WHO description, MFH of bone is a highly aggressive primary bone tumor of unknown cellular origin. The tumor has a rather typical and distinct clinical presentation as a lytic destructive lesion, affecting adults and showing a predilection for the long bones of the lower extremities. Histologically, it is characterized by a mixture of spindle-shaped, histiocytoid and pleomorphic cells. Atypical nuclei and mitoses are common, and multinucleated tumor and osteoclastic giant cells and inflammatory cells are often present. There are no specific immunohistochemical markers, and it may thus be difficult to distinguish it from other bone tumors with scarce or no osteoid formation, such as fibrosarcoma and poorly differentiated osteosarcoma [5].

In an attempt to evaluate whether a reclassification of MFH of bone, similar to that for soft tissue tumors, is possible, 57 tumors classified as MFH of bone were reviewed by a panel of expert bone pathologists from the EuroBoNeT network, a network of excellence studying the pathology and genetics of bone tumors [Romeo et al: Malignant fibrous histiocytoma and fibrosarcoma of bone in 2011: What's new? Submitted]. Of these, 11 had been subjected to chromosome banding analysis after short-term culturing. In the present study, we report the cytogenetic findings, and compare the karyotypic features with those of the most important differential diagnoses.

## Methods

### Patients

Clinical data are summarized in Table 1. Case numbers correspond to those in the article by Romeo et al. [Malignant fibrous histiocytoma and fibrosarcoma of bone in 2011: What's new? Submitted], in which the morphologic and immunohistochemical features of a larger series of so-called MFH of bone are detailed. In brief, the present study included six men and five women, aged 29-76 years at diagnosis. All tumors were primary lesions; from Case 91 also a lung metastasis could be analyzed. Tumor locations were lower (n = 6) and upper (2) extremities, pelvis (2), and unknown (1).

**Table 1:** Karyotypes of cytogenetically abnormal tumors previously diagnosed as MFH of bone.

**Case No.**^**1**^	Age/Sex	**Diagnosis**^**2**^	Site	**Karyotype**^**3**^	**FISH**^**4**^
Case 63 + 64	61/M	Myoepithelioma-like sarcoma	Humerus	42, XY, der(1)t(1;10)(p11;q11), +dic(1;15)(p13;q26), add(6)(p11),-7, add(7)(p22), t(8;9)(q22;p24),-10, add(10)(q11), add(12)(q24),-15, +add(16)(p13),-17, add(19)(p13),-20, -20, r(21)(p13q22), r(22)(p13q13), +1-2mar	*EWSR1-*, *FUS-*
Case 69	49/M	Myoepithelioma-like sarcoma	Tibia	77-94 < 4n >, X,-X, del(X)(q21),-Y, add(1) (q25), del(2)(p14)x2,-3,-4, der(4)t(4;5) (q?31;q?15),-5,-5,-6,-8,-8,-9,-9,-9,-9, -10,-10, add(11)(q13)x2, add(12) (q22)x2,-13,-15,-16, add(16)(p13),-17, -17,-17,-17,+18,-19, add(20)(p13)x4, -21,-21, inc	*EWSR1-*, *FUS-*
Case 88	70/F	Myoepithelioma-like sarcoma	Femur	54-58 < 2n >, XX,+der(1;9)(q10;q10), +i(5)(p10),+add(6)(q13),+7,+del(8) (q21-22)x2,-9,+15,+20,+20,+20,+22, +22,+3-4 mar	Failure
Case 89	53/M	Leiomyosarcoma	Femur (bone infarct)	58-61, XX,-Y,+i(1)(q10),-3,-4,-5,-6, +add(7)(q11), add(9)(p21)x2,-10,-10, -11, i(11)(p10),-12, add(12)(p13)x2, -13,-14, i(15)(q10),-16,-17,-17, add(19) (q13),+20,+i(20)(p10),-21, der(21) t(1;21)(p13;p11),-22,+2-4mar	ND
Case 98^5^	47/M	Leiomyosarcoma	Os ileum	130-190 < 6n-8n >, X?inc	ND
Case 68	76/F	UPS with incomplete myogenic differentiation	Femur	59-113, X?, inc/46, XX, inv(9)(p11q12)c	ND
Case 66	68/F	UPS	Tibia	84-106 < 5n >, XX,-X,-X,-X,?add(1) (q42), del(1)(p11), del(1)(q12), der(1) add(1)(q42)del(1)(p11), der(1)add(1) (q44)add(1)(p11)hsr(?), add(2)(q3?3), i(3)(p10),-4, der(4)add(4)(p16)hsr(4) (p16), i(5)(q10),+add(6)(q11)x2,-7, der(8)t(1;8)(p22;p2?3)x1-2, i(10)(q10), -13,-13, add(13)(p11),-14, add(14)(p11), ?i(14)(q10),-15,-15,-16,-16,-17,-17, ?add(17)(p11),-18,-18, der(18)add(18) (p11)hsr(?),-19,-21,-21,-22,?add(22) (q13), inc	ND
Case 105	66/F	UPS	Unknown	67-77, XX,-X,+1,-2, add(2)(p11)x2,-3, +4,-8,-8,+9, dic(9;17)(p21;p13)x2,+10, +12,+13, der(14)t(2;14)(q11;p11),+16, +17,+19,-20,-22	ND
Case 100	52/F	Osteosarcoma	Os ischium	32-44, X,-X, add(1)(p11),-3,-4,-6,-9,-10, der(12)add(12)(p12)del(12)(q22),-13, -13,-14,-15,-16,-17,-18,-19,-21, add(21) (p11),-22, der(22)t(3;22)(p11;q11)	ND
Case 86 + 94	51/M	SCS NOS (metastatic carcinoma?)	Femur	44-48, XY, der(1)t(1;18)(p36;q11), del(4)(p15), del(7)(q22), del(8)(p11), dic(8;10)(p11;p?),-13,-15, del(16)(q22), add(19)(p13), add(20)(q13),+add(21) (q22),+r,+1-7mar/80-83, idemx2	ND
Case 91	29/M	Dubious biphasic sarcoma	Humerus Lung met.	47, XY, add(19)(q13),+mar 88-90, XXYY, der(2)t(2;7)(p21;q11)x2, -4,-4,+i(5)(p10)x2,-7,-7,-12, del(12) (q22)x2, add(13)(q22)x2,+18,-21,-21, +3-4 mar	ND

^1^Case numbers are identical to those in the publication by Romeo et al. [6].

^2^All tumors were classified as grade 3 tumors (3-grade scale). UPS = undifferentiated pleomorphic sarcoma; SCS NOS = spindle cell sarcoma not otherwise specified.

^3^All karyotypes are composite karyotypes.

^4^ND = not done.

^5^Karyotype on sample obtained after chemotherapy.

### Histopathologic examination

The 11 tumors were originally diagnosed as MFH of bone, but were all reclassified and subtyped as described elsewhere [Romeo et al: Malignant fibrous histiocytoma and fibrosarcoma of bone in 2011: What's new? Submitted] by a board of pathologists (S.R., J.V.M.G.B., R.T., N.A., P.C.W.H., A.P.D.T.). Three tumors were classified as myoepithelioma-like sarcoma, two as leiomyosarcoma, one as UPS with incomplete myogenic differentiation, two as UPS, one as osteosarcoma, one as spindle cell sarcoma not otherwise specified, and one as an unclassifiable biphasic sarcoma (Table 1). All tumors were classified as high-grade (grade 3) lesions.

### Chromosome banding analysis

Fresh tumor samples were processed for G-banding analysis as described [6], and karyotypes were described according to the guidelines in ISCN 2009 [7].

### Fluorescence in situ hybridization (FISH)

FISH using break-apart probes for the *EWSR1 *and *FUS *genes was performed on interphase nuclei in cut sections from paraffin-embedded tumors reclassified as myoepithelioma-like sarcomas. For details, see Romeo et al. [Malignant fibrous histiocytoma and fibrosarcoma of bone in 2011: What's new? Submitted].

## Results

The karyotypes, which were based on G-banding alone, were highly complex in all cases (Table 1). The only exception was the primary lesion of Case 91, showing addition of unknown material to the long arm of chromosome 19 and a supernumerary marker chromosome as the sole changes; however, the lung metastasis of the same tumor showed an unrelated highly complex karyotype. The chromosome number varied from 32 to approximately 190, with a near-diploid or near-triploid modal chromosome number in five and three cases, respectively. In all cases there were aberrations that could not be resolved, resulting in karyotypes with chromosomes with material added from an unknown chromosome and/or multiple marker chromosomes. In spite of the large number of structural rearrangements, only a few breakpoints were recurrent: 1p11, the centromeric region of chromosome 5 and 12q22 were each affected in three cases, and involvement of 1p13, the centromeric region of chromosome 1, 7q11, 9p21, 14p11, 16p13, 19p13, 19q13, 21p11, 21q22 and 22q13 was seen in two cases each. A total of 68 different chromosome bands were involved in structural rearrangements; two-thirds of these were near-centromeric (p11-q11; 26 bands) or terminal (19 bands) chromosome bands. The only breakpoints which were recurrent in, and restricted to, a particular morphologic subtype were 14p11 in UPS and 16p13 in myoepithelioma-like sarcomas. Bearing in mind the incompleteness of the karyotypic descriptions, no attempts were made to identify chromosomal imbalances resulting from numerical or unbalanced structural rearrangements. Only one balanced translocation, a t(8;9)(q22;p24) in a myoepithelioma-like sarcoma, was found. Neither this aberration, nor any of the other unbalanced translocations or deletions corresponded to any known tumor-specific rearrangement. Cytogenetic signs of gene amplification, in the form of double minutes, homogeneously staining regions or ring chromosomes, were seen in two cases.

Interphase FISH analysis for rearrangement of the *EWSR1 *and *FUS *loci was successfully performed in two of the three cases of myoepithelioma-like sarcoma. Neither case showed a rearrangement.

## Discussion

The genetic information on so-called MFH of bone is limited. In a series of 19 cases, *TP53 *mutations were found in two, whereas the *CDKN2A *gene was not affected [8]. Another 26 cases were analyzed by chromosome-based comparative genomic hybridization (CGH), revealing copy number changes in 23 of them [9]. The results indicated a different pattern of chromosomal imbalances compared to that in high-grade osteosarcomas, fibrosarcomas of bone, and soft tissue MFHs. Gains were more common than losses, with approximately one-third of the cases displaying gain of material from chromosome arms 1q, 7p, 7q, 8q, 9q, or 15q [9]. Whereas CGH analyses are more reliable in providing information on copy number changes, chromosome banding analyses have the advantage of identifying balanced chromosomal exchanges, as well as revealing the ploidy level and the extent of intercellular variation. However, only five cases with abnormal karyotypes have previously been reported, all presenting complex karyotypes without any obvious unifying feature [6,10-13].

The results of the present study add to the conclusion that tumors previously recognized as MFH of bone are genetically highly complex, and that there are few recurrent aberrations that can be detected by chromosome banding analysis. Neither the overall pattern of chromosome aberrations (aneuploidy, high level of complexity or intercellular variation) nor specific findings (e.g., particular breakpoints or aberrations) distinguish these tumors cytogenetically from their main differential diagnostic entity - osteosarcoma.

The present study included too few cases of each subtype to allow any meaningful comparison between the new morphologic subgroups; until more cases have been analyzed, the finding of two recurrent breakpoints in UPS (14p11) and myoepithelioma-like sarcoma (16p13) should be considered chance findings. One of the morphologic subgroups identified at the histopathologic re-evaluation - myoepithelioma-like sarcoma - could potentially harbor a distinctive genetic aberration. Antonescu and co-workers recently showed that gene fusions involving the *EWSR1 *gene are common in myoepithelial tumors of soft tissue and bone [14]. An *EWSR1/POU5F1*, *EWSR1/PBX1 *or *EWSR1/ZNF444 *fusion gene, or a rearrangement of the *EWSR1 *gene with unknown fusion partner, was detected in close to half of the soft tissue lesions, and in four out of five bone tumors. However, all *EWSR1*-positive bone tumors were classified as benign; the single malignant myoepithelial tumor of bone was negative. However, one of the seven sarcomas analyzed in the study by Romeo et al. [Malignant fibrous histiocytoma and fibrosarcoma of bone in 2011: What's new? Submitted] - reclassified as myoepthelioma-like sarcoma - had an *EWSR1/NFATC2 *fusion, adding to the growing morphological spectrum of this recently described gene fusion [15]. In the present study, only two of the cases diagnosed as myoepithelial-like sarcomas could be analyzed by FISH for *EWSR1*-rearrangement, and both were negative. Combined, these data indicate that *EWSR1*-rearrangements are rare in malignant bone tumors showing myoepithelial differentiation.

Based on the present, admittedly small, study it seems safe to conclude that traditional chromosome banding analysis cannot be used to distinguish between the many different morphologic subtypes that may be discerned among so-called MFH of bone. Nor is there any indication that the karyotypic features of any of the subgroups differ from those in osteosarcoma. Quite possibly, also high resolution, array-based genomic analyses will fail to detect any distinct features, as previously shown for soft tissue tumors [16], but that needs to be properly evaluated in larger series.

## Competing interests

The authors declare that they have no competing interests.

## Authors' contributions

FM, SR, PCWH, APDT. and MD-R. designed the study and wrote the manuscript. FM, SR, JVMB, RT, NA, MA, RS, HAD, KÅ and NM. performed histopathological, cytogenetic and/or FISH studies. All autors read and approved the final version of the manuscript.
